# Supplementary material for: Hot spot prediction in protein-protein interactions by an ensemble system
Source: BMC Syst Biol. 2018 Dec 31;12(Suppl 9):132. doi: 10.1186/s12918-018-0665-8 (PMC6311905; doi:10.1186/s12918-018-0665-8)
Supplement: Supplementary file 2 — All relevant MATLAB code. A simple Matlab implement of our predictor. (ZIP 19,742 kb) [file 12918_2018_665_MOESM2_ESM.zip › Additional file 2/Read ASA/ASA website/dbMPIKT/4HSAA.html]

 NetSurfP - Protein Surface Accessibility and Secondary Structure Predictions

  

|  |  |  |
| --- | --- | --- |
|  |  | NetSurfP - Protein Surface Accessibility and Secondary Structure Predictions  Technical University of Denmark |

  


---

```
# For publication of results, please cite:
# A generic method for assignment of reliability scores applied to solvent accessibility predictions.
# Bent Petersen, Thomas Nordahl Petersen, Pernille Andersen, Morten Nielsen and Claus Lundegaard
# BMC Structural Biology 2009, 9:51 doi:10.1186/1472-6807-9-51
#
# Column 1: Class assignment - B for buried or E for Exposed - Threshold: 25% exposure, but not based on RSA
# Column 2: Amino acid
# Column 3: Sequence name
# Column 4: Amino acid number
# Column 5: Relative Surface Accessibility - RSA
# Column 6: Absolute Surface Accessibility
# Column 7: Z-fit score for RSA prediction
# Column 8: Probability for Alpha-Helix
# Column 9: Probability for Beta-strand
# Column 10: Probability for Coil
E P  4HSAA                  1    0.725 102.821  -1.093   0.003   0.003   0.994
E N  4HSAA                  2    0.688 100.665  -0.181   0.018   0.019   0.964
E S  4HSAA                  3    0.548  64.190  -0.906   0.115   0.016   0.868
E E  4HSAA                  4    0.667 116.472  -0.688   0.115   0.016   0.868
E D  4HSAA                  5    0.664  95.697  -0.671   0.058   0.017   0.925
E K  4HSAA                  6    0.579 119.203  -1.594   0.058   0.017   0.925
E N  4HSAA                  7    0.588  86.054  -0.400   0.058   0.017   0.925
B F  4HSAA                  8    0.133  26.673  -0.141   0.115   0.016   0.868
B P  4HSAA                  9    0.255  36.213  -0.241   0.113   0.043   0.844
E R  4HSAA                 10    0.410  93.936  -0.212   0.199   0.152   0.649
B T  4HSAA                 11    0.203  28.225  -0.302   0.148   0.418   0.435
B V  4HSAA                 12    0.057   8.746  -0.455   0.085   0.775   0.140
B M  4HSAA                 13    0.229  45.823   0.584   0.018   0.846   0.136
B V  4HSAA                 14    0.066  10.206  -0.320   0.021   0.756   0.223
B N  4HSAA                 15    0.216  31.652  -0.077   0.023   0.655   0.322
B L  4HSAA                 16    0.102  18.640  -0.109   0.021   0.451   0.528
E N  4HSAA                 17    0.361  52.836  -0.130   0.069   0.386   0.545
B I  4HSAA                 18    0.191  35.409  -1.173   0.074   0.484   0.442
B H  4HSAA                 19    0.255  46.366  -1.179   0.074   0.484   0.442
E N  4HSAA                 20    0.324  47.478  -1.542   0.066   0.296   0.638
B R  4HSAA                 21    0.281  64.326  -0.655   0.064   0.216   0.721
E N  4HSAA                 22    0.426  62.352  -1.899   0.018   0.088   0.893
E T  4HSAA                 23    0.379  52.512  -1.543   0.018   0.047   0.935
E N  4HSAA                 24    0.561  82.086  -1.365   0.018   0.047   0.935
E T  4HSAA                 25    0.584  81.029  -0.749   0.018   0.088   0.893
E N  4HSAA                 26    0.572  83.814  -1.983   0.018   0.088   0.893
E P  4HSAA                 27    0.394  55.965  -1.810   0.018   0.088   0.893
E K  4HSAA                 28    0.561 115.398  -1.540   0.018   0.088   0.893
E R  4HSAA                 29    0.401  91.898  -0.792   0.019   0.141   0.840
E S  4HSAA                 30    0.661  77.446  -1.175   0.018   0.088   0.893
E S  4HSAA                 31    0.438  51.357  -1.254   0.018   0.047   0.935
E D  4HSAA                 32    0.525  75.667   0.072   0.018   0.088   0.893
B Y  4HSAA                 33    0.189  40.453  -0.534   0.056   0.142   0.802
E Y  4HSAA                 34    0.412  88.151  -0.160   0.056   0.142   0.802
E D  4HSAA                 35    0.496  71.445  -0.260   0.056   0.142   0.802
B R  4HSAA                 36    0.215  49.235  -0.417   0.056   0.142   0.802
B S  4HSAA                 37    0.140  16.408  -1.695   0.020   0.205   0.775
B T  4HSAA                 38    0.120  16.644  -0.825   0.020   0.205   0.775
B S  4HSAA                 39    0.115  13.466   0.094   0.019   0.141   0.840
B P  4HSAA                 40    0.126  17.908  -1.481   0.019   0.141   0.840
B W  4HSAA                 41    0.164  39.490  -0.665   0.021   0.451   0.528
E N  4HSAA                 42    0.351  51.342  -0.301   0.022   0.552   0.426
B L  4HSAA                 43    0.165  30.248   0.323   0.023   0.655   0.322
E H  4HSAA                 44    0.412  75.034   0.325   0.021   0.756   0.223
B R  4HSAA                 45    0.161  36.915   0.531   0.022   0.552   0.426
E N  4HSAA                 46    0.340  49.747  -0.247   0.021   0.279   0.699
E E  4HSAA                 47    0.463  80.939   0.084   0.019   0.141   0.840
E D  4HSAA                 48    0.318  45.867  -0.464   0.018   0.047   0.935
E P  4HSAA                 49    0.502  71.262  -0.657   0.018   0.019   0.964
E E  4HSAA                 50    0.550  95.998  -1.482   0.058   0.017   0.925
B R  4HSAA                 51    0.257  58.899   0.256   0.113   0.043   0.844
B Y  4HSAA                 52    0.180  38.445   0.967   0.184   0.043   0.773
B P  4HSAA                 53    0.099  14.062  -0.188   0.268   0.043   0.689
E S  4HSAA                 54    0.349  40.926   0.127   0.455   0.046   0.498
E V  4HSAA                 55    0.291  44.773   0.683   0.660   0.049   0.291
B I  4HSAA                 56    0.058  10.637   1.197   0.751   0.050   0.199
B W  4HSAA                 57    0.138  33.141   0.118   0.831   0.044   0.125
E E  4HSAA                 58    0.256  44.706   0.050   0.751   0.050   0.199
B A  4HSAA                 59    0.029   3.152  -0.481   0.751   0.050   0.199
E K  4HSAA                 60    0.278  57.226   0.896   0.701   0.107   0.192
B C  4HSAA                 61    0.031   4.352  -0.261   0.538   0.173   0.289
B R  4HSAA                 62    0.204  46.785   0.594   0.453   0.248   0.299
B H  4HSAA                 63    0.097  17.699  -0.164   0.321   0.252   0.427
E L  4HSAA                 64    0.429  78.477   1.462   0.118   0.150   0.732
E G  4HSAA                 65    0.326  25.648  -1.398   0.052   0.084   0.864
B C  4HSAA                 66    0.043   5.967  -1.061   0.052   0.084   0.864
B I  4HSAA                 67    0.142  26.270   0.190   0.018   0.088   0.893
B N  4HSAA                 68    0.281  41.138  -0.264   0.018   0.047   0.935
E A  4HSAA                 69    0.366  40.355  -1.351   0.018   0.019   0.964
E D  4HSAA                 70    0.582  83.881  -0.902   0.058   0.017   0.925
E G  4HSAA                 71    0.281  22.138  -1.424   0.053   0.043   0.903
E N  4HSAA                 72    0.469  68.706  -0.446   0.064   0.216   0.721
E V  4HSAA                 73    0.326  50.137  -1.024   0.069   0.386   0.545
E D  4HSAA                 74    0.368  52.986  -0.751   0.066   0.296   0.638
E Y  4HSAA                 75    0.333  71.098  -0.093   0.021   0.279   0.699
E H  4HSAA                 76    0.339  61.610  -0.679   0.020   0.205   0.775
B M  4HSAA                 77    0.201  40.300  -0.230   0.022   0.359   0.619
E N  4HSAA                 78    0.359  52.528  -0.616   0.021   0.451   0.528
B S  4HSAA                 79    0.110  12.869  -1.176   0.022   0.552   0.426
B V  4HSAA                 80    0.213  32.692   0.300   0.023   0.655   0.322
B P  4HSAA                 81    0.162  23.030  -0.274   0.022   0.552   0.426
B I  4HSAA                 82    0.071  13.117   0.236   0.023   0.655   0.322
E Q  4HSAA                 83    0.279  49.812  -0.014   0.021   0.756   0.223
B Q  4HSAA                 84    0.224  40.042   0.514   0.004   0.616   0.381
E E  4HSAA                 85    0.502  87.787   0.438   0.004   0.616   0.381
B I  4HSAA                 86    0.085  15.651   0.470   0.003   0.718   0.279
B L  4HSAA                 87    0.203  37.188   0.437   0.018   0.846   0.136
B V  4HSAA                 88    0.046   7.039  -0.438   0.011   0.918   0.071
B L  4HSAA                 89    0.118  21.624   0.054   0.011   0.918   0.071
B R  4HSAA                 90    0.214  49.006   0.323   0.018   0.846   0.136
B R  4HSAA                 91    0.242  55.487  -0.394   0.004   0.616   0.381
E E  4HSAA                 92    0.512  89.429   0.216   0.005   0.262   0.733
E P  4HSAA                 93    0.545  77.335  -1.142   0.004   0.138   0.858
E P  4HSAA                 94    0.653  92.647  -1.040   0.005   0.045   0.951
E H  4HSAA                 95    0.691 125.638  -1.199   0.005   0.015   0.979
B S  4HSAA                 96    0.275  32.265  -0.905   0.005   0.015   0.979
E P  4HSAA                 97    0.513  72.781  -0.281   0.004   0.085   0.910
B N  4HSAA                 98    0.291  42.661  -0.209   0.005   0.336   0.660
E S  4HSAA                 99    0.276  32.359   0.317   0.004   0.616   0.381
B F  4HSAA                100    0.084  16.859   0.568   0.002   0.816   0.182
E R  4HSAA                101    0.302  69.112   0.943   0.001   0.900   0.099
E L  4HSAA                102    0.283  51.744   0.481   0.001   0.900   0.099
B E  4HSAA                103    0.236  41.247   0.017   0.001   0.900   0.099
E K  4HSAA                104    0.323  66.503   0.407   0.001   0.900   0.099
B I  4HSAA                105    0.161  29.766   0.668   0.001   0.959   0.040
E L  4HSAA                106    0.373  68.370   0.846   0.001   0.959   0.040
B V  4HSAA                107    0.054   8.377   1.235   0.001   0.900   0.099
B S  4HSAA                108    0.179  20.991   0.118   0.002   0.816   0.182
B V  4HSAA                109    0.064   9.821   0.044   0.003   0.718   0.279
B G  4HSAA                110    0.088   6.910  -0.802   0.004   0.616   0.381
B C  4HSAA                111    0.037   5.251   0.211   0.023   0.655   0.322
B T  4HSAA                112    0.129  17.851   0.306   0.018   0.846   0.136
B C  4HSAA                113    0.039   5.490   0.142   0.011   0.918   0.071
B V  4HSAA                114    0.063   9.714   0.275   0.021   0.756   0.223
B T  4HSAA                115    0.172  23.870   1.023   0.074   0.484   0.442
B P  4HSAA                116    0.165  23.399  -0.164   0.159   0.516   0.325
B I  4HSAA                117    0.206  38.054   0.832   0.148   0.418   0.435
B V  4HSAA                118    0.180  27.651  -0.968   0.159   0.516   0.325
E H  4HSAA                119    0.399  72.524   0.129   0.169   0.612   0.219
E H  4HSAA                120    0.483  87.912  -0.432   0.252   0.423   0.325
E V  4HSAA                121    0.438  67.397  -0.406   0.191   0.086   0.723
E A  4HSAA                122    0.900  99.125  -0.712   0.003   0.003   0.994

Explain the output.  Go back.
```

---
